# Malignant Pleural Mesothelioma: From Pathophysiology to Innovative Actionable Targets

**DOI:** 10.3390/cancers17071160

**Published:** 2025-03-30

**Authors:** Francesco Rocco Bertuccio, Simone Montini, Maria Antonietta Fusco, Antonella Di Gennaro, Gaetano Sciandrone, Francesco Agustoni, Giulia Galli, Chandra Bortolotto, Jessica Saddi, Guido Baietto, Giulio Melloni, Gioacchino D’Ambrosio, Angelo Guido Corsico, Giulia Maria Stella

**Affiliations:** 1 Department of Internal Medicine and Medical Therapeutics, University of Pavia Medical School, 27100 Pavia, Italy; f.bertuccio@smatteo.pv.it (F.R.B.); simone.montini@universitadipavia.it (S.M.); m.fusco@smatteo.pv.it (M.A.F.); a.digennaro@smatteo.pv.it (A.D.G.); g.sciandrone@smatteo.pv.it (G.S.); f.agustoni@smatteo.pv.it (F.A.); g.galli@smatteo.pv.it (G.G.); a.corsico@smatteo.pv.it (A.G.C.); 2 Unit of Respiratory Diseases, Cardiothoracic and Vascular Department, IRCCS Policlinico San Matteo, 27100 Pavia, Italy; 3 Department of Medical Oncology, Fondazione IRCCS Policlinico San Matteo, 27100 Pavia, Italy; 4 Diagnostic Imaging and Radiotherapy Unit, Department of Clinical, Surgical, Diagnostic and Pediatric Sciences, University of Pavia, 27100 Pavia, Italy; c.bortolotto@smatteo.pv.it; 5 Radiology Institute, Fondazione IRCCS Policlinico San Matteo, 27100 Pavia, Italy; 6 Unit of Radiation Therapy, Department of Oncology, Clinical-Surgical, IRCCS Policlinico San Matteo Foundation, 27100 Pavia, Italy; j.saddi@smatteo.pv.it; 7 Department of Radiation Oncology, Fondazione IRCCS Policlinico San Matteo, 27100 Pavia, Italy; 8 Unit of Thoracic Surgery, Cardiothoracic and Vascular Department, IRCCS Policlinico San Matteo, 27100 Pavia, Italy; g.baietto@smatteo.pv.it (G.B.); g.melloni@smatteo.pv.it (G.M.); 9 Pathology Unit, Department of Diagnostical Services and Imaging, Fondazione IRCCS Policlinico San Matteo, 27100 Pavia, Italy; g.dambrosio@smatteo.pv.it

**Keywords:** pleural mesothelioma, actionable targets, genetics, personalized medicine

## Abstract

Background: Pleural mesothelioma (PM) is a rare and highly aggressive cancer which arises from mesothelial layer and primarily linked to asbestos exposure, genetic predispositions, and specific mutations. Despite current treatment modalities, including chemotherapy, antiangiogenic therapy and more recently immunotherapy, the prognosis remains dismal, with a median survival time of 6–18 months. Objectives: The urgent need for novel therapeutic strategies has prompted research into molecular targets and precision medicine approaches. At present, many potential targets for therapeutic strategies have been identified, and emerging clinical trials are demonstrating certain clinical efficacy. Methods: This review examines advancements in understanding PM’s genetic and epigenetic landscape, signaling pathways, and promising therapeutic targets. Results: We also discuss the results of recent clinical trials and their potential implications for future treatment paradigms.

## Simple Summary

Pleural mesothelioma is a malignant tumor that originates from the serous membrane, the mesothelium, which lines the pleural space. In addition to being a rare and orphan disease, actionable and treatable traits of the disease are poorly understood and exploited. A deeper knowledge regarding the pathogenic mechanisms of the disease is mandatory to point out how to develop innovative and really efficient diagnostic and therapeutic approaches. Thus, the aim of this review that is namely to summarize and discuss the state-of-the art and the validated data on PM biologic features and discuss the most promising and reliable treatment perspectives.

## 1. Introduction

Pleural mesothelioma (PM) is a devastating malignancy originating from mesothelial cells of the pleural lining. Its etiology is strongly associated with exposure to asbestos, a known carcinogen, which triggers chronic inflammation, DNA damage, and carcinogenesis. Inhalation of asbestos fibers induces mesothelial inflammation, the clustering of immune cells, and the activation of carcinogenic pathways, eventually leading to malignancy. Beyond asbestos exposure, genetic factors and mutations, such as those in *BAP1*, *TP53*, *CDKN2A*, and *NF2* genes play critical roles in PM pathogenesis [1]. PM predominantly affects individuals with a history of occupational asbestos exposure, and recent data from the International Agency for Research on Cancer indicate 30,618 new cases and 25,372 deaths globally in 2022 [2]. The World Health Organization (WHO) classifies PM into three main histological subtypes: epithelioid, biphasic, and sarcomatoid, with epithelioid being the most common. Most patients are diagnosed at advanced stages, resulting in poor survival outcomes. The five-year survival rate remains below 7%, with a median overall survival (mOS) of 6–12 months when treated with first-line pemetrexed and carboplatin (PemC) [3]. Concerning the surgical approach, over the years, it has proved to have limited benefits. The MARS trial, which was published in 2011, sought to determine how extra-pleural pneumonectomy (EPP) affected the quality of life and survival of patients with malignant pleural mesothelioma [4]. Despite their limitations, our data indicated that EPP in trimodal therapy is not helpful and may even be harmful to patients [5,6]. Additionally, the much-awaited results of the MARS2 clinical trial, which compared the survival of two groups of patients with pleural mesothelioma—one group receiving chemotherapy and surgery (extended pleurectomy decortication) and the other receiving chemotherapy without surgery—were presented by researchers in October 2023. The survival times of the two groups did not differ significantly, according to the results [7].

**Figure 1:**
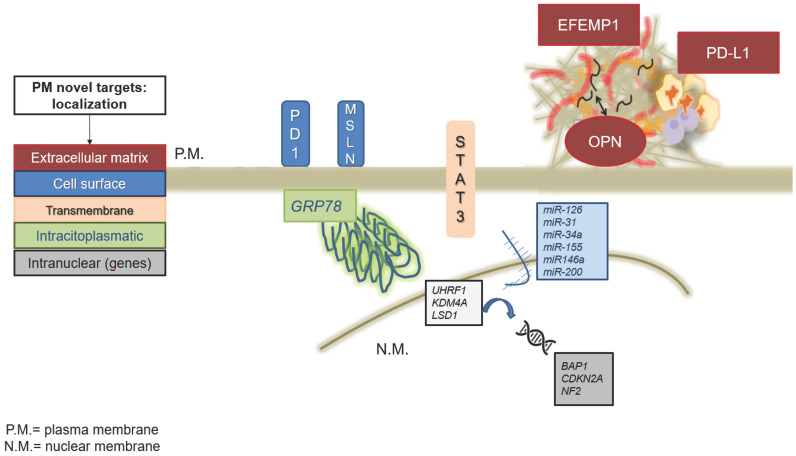
PM novel targets and their localization.

While additional therapies, such as bevacizumab and immune checkpoint inhibitors (e.g., nivolumab and ipilimumab), have modestly improved survival, they are far from curative. Notably, the DREAM II trial demonstrated that combining PemC with the PD-L1 inhibitor durvalumab resulted in a median overall survival (mOS) of 18.4 months, and the 12-month survival rate was 65%. Despite these advances, the effectiveness of standard therapies remains limited, underscoring the urgent need for innovative treatments [8]. Recent advancements in high-throughput technologies and -OMIC platforms have shed light on PM’s molecular underpinnings, unveiling novel therapeutic targets (Figure 1). These include epigenetic regulators, signaling proteins, and aberrant gene pathways. The biomolecular heterogeneity of PM, characterized by the absence of dominant genetic drivers, has historically hindered the development of targeted therapies. However, this heterogeneity is now being leveraged to identify and exploit unique vulnerabilities in PM’s molecular assets. Emerging preclinical studies have highlighted the interplay of key pathways such as PI3K/Akt, MAPK, and Hippo, which are deregulated in PM. These pathways contribute to the aggressive behavior of the disease, including resistance to apoptosis, unchecked proliferation, and enhanced invasion. Furthermore, alterations in epigenetic modifiers such as EZH2, UHRF1, and LSD1 underscore the potential of targeting chromatin remodeling mechanisms to halt tumor progression [9]. This review explores recent progress in understanding PM’s molecular pathobiology, including signal transduction pathways, genetic mutations, and epigenetic modifications. We also examine the advancements in therapeutic strategies and ongoing clinical trials aimed at improving the outcomes for MPM patients.

### 1.1. Glucose-Regulated Protein 78 (GRP78)

Protein folding and assembly depend on GRP78, an endoplasmic reticulum chaperone protein sometimes referred to as binding protein (BIP), and heat-shock protein 5A (HSP5A). PM and other malignancies have been found to display high GRP78 levels, which encourage tumor development, chemoresistance, and survival [10]. The Unfolded Protein Response (UPR) is an adaptive mechanism that is triggered in response to cellular stress in order to restore cellular homeostasis. PERK, ATF6, and IRE1 are the three UPR branches whose activation is regulated by GRP78. By associating with GRP78, these sensors are rendered inactive; nevertheless, when misfolded proteins build up in the endoplasmic reticulum (ER) lumen, GRP78 separates, causing these sensors to become active [11]. In order to reestablish protein synthesis balance, each arm of the UPR will in turn trigger a transcription factor that will start a cell survival response [12,13]. Previously linked to the tumor’s aggressiveness and growth, GRP78 expression and protein levels are elevated in a number of cancer types. By binding to caspases and preventing their activation, GRP78 increases cells’ resistance to cell death. Although it can be present in other compartments like the mitochondria and nucleus, GRP78 is mostly located in the ER. When secreted, it can also be found in the peripheral circulation or in the cell membrane [14,15]. GRP78, also known as CELL-SURFACE (CS-GRP78), can be transferred from the ER to the cell’s plasma membrane [12,13]. GRP78 functions as a receptor and co-receptor for soluble ligands and is suggested as a potential signal transducer of tumor cells through its interactions with several membrane proteins. Cell signaling, inflammation, proliferation, invasion, apoptosis, and immunology all appear to be significantly impacted by CS-GRP78. GRP78 overexpression in cancer cells suggests that this chaperone could be a highly effective predictive biomarker for several cancer types [16]. When defining treatment, a prognostic biomarker may be crucial since it indicates the probability of disease, recurrence, or progression. GRP78 may help guide treatment choices if it is a biomarker of tumor aggressiveness [17]. By raising oxidative stress and upsetting calcium homeostasis, BOLD-100, a GRP78 modulator, has shown lethal effects on PM cells, resulting in unfolded protein response (UPR)-mediated death [18]. Early-phase clinical trials have demonstrated longer progression-free survival (mPFS) and mOS in different malignancies, and in vitro experiments have demonstrated that BOLD-100 dramatically suppresses PM cell proliferation (Table 1). Overall, further investigation regarding GRP78-targeted treatments in PM is required [19,20].

### 1.2. Fibulin-3 (EFEMP1)

An extracellular matrix protein called fibrin-3 is overexpressed in PM and is associated with cancer development and aggressiveness. Patients with PM have been distinguished from those with benign pleural diseases by elevated levels of pleural effusions. Fibulin-3 promotes cell invasion and proliferation by activating the PI3K/Akt signaling pathway. Fibulin-3 is a promising biomarker and therapeutic target because experimental models show that it greatly lowers tumor burden (Table 1) [21,22]. The observed reduction in cell proliferation and migration/invasion was matched by the molecular disruption of cell adhesion and cell-ECM interaction pathways caused by fibulin-3 knockdown in MPM cells. Additionally, pathway analysis showed that PI3K/Akt signaling regulates a number of the cell-adhesion and motility genes that were downregulated in cells lacking fibulin-3. While fibulin-3 overexpression raised both PI3K and Akt activation, fibulin-3 knockdown decreased both MAPK and PI3K/Akt phosphorylation [23,24]. This is in line with fibulin-3’s function as an organizer of the extracellular matrix scaffold that surrounds tumor cells and implies that it controls a PI3K/Akt/MAPK cascade that initiates PM cell proliferation and motility. More significantly, fibulin-3’s boosting effect on PI3K-dependent gene expression and PM cell viability is eliminated by a PI3K inhibitor, indicating that PI3K activation is required for the effects of this ECM protein. According to these findings, fibulin-3 is adequate to trigger PI3K/Akt signaling in PM cells, and it is dependent upon this process to provide its pro-tumoral effects in PM [25,26]. The functional connection between fibulin-3 overexpression in PM and tumor aggressiveness, despite the fact that the overall mechanism of fibulin-3 in PM is still unclear, increases the protein’s significance as a tumor biomarker and possible tumor target. Animal tests using a function-blocking antibody that stops fibulin-3 from signaling in PM cells were conducted in order to further confirm that fibulin-3 is a clinically relevant target in PM. This antibody is a mouse monoclonal antibody (mAb428.2) that has been chimerized into a human [21,27]. By blocking these putative signaling cascades, treating PM with the chimera mAb428.2 decreased tumor development and suppressed the proliferation of malignant cells. Significantly, the number of long-term surviving animals and the median survival both increased when tumor progression was halted during mAb428.2 treatment and only began long after the treatment ended. This implies that the duration of our therapy and the availability of antibodies are the only factors limiting the long-term tumor-suppressive efficacy of fibulin-3 inhibition in PM cells, even in cell types that secrete high levels of this protein [27].

### 1.3. Signal Transducer and Activator of Transcription-3 (STAT-3)

In PM, STAT-3 is constitutively activated and mediates immune evasion, inflammation, cell survival, and proliferation. While phosphorylation at serine 727 modifies the function of STAT-3, phosphorylation at tyrosine 705 causes nuclear translocation and gene transcription. Under physiological settings, the transcription factor STAT-3 controls the expression of genes governing survival, proliferation, and self-renewal and is momentarily activated in response to cytokines and growth stimuli. The signal transducer and activator of transcription 3 (STAT-3) is constitutively activated in a large number of solid tumors and hematologic malignancies, which propels the tumor cells’ malignant activity [28,29]. Of the PM cases that were archived, 61.4% (27/44) had activated tyrosine phosphorylated STAT3. The precise function of STAT3 in PM is unclear, though. Chemotherapy-resistant PM cell lines have a physical and functional connection between NFkB and STAT3, and chemoresistance may depend on the stability of the STAT3–NFkB complex. Furthermore, higher STAT3 activation and poor survival in PM are linked to low expression levels of Protein Inhibitor of Activated STAT3 (PIAS3), an endogenous inhibitor of STAT3 signaling. Mutations in tumor suppressor genes, which are unrelated to STAT3 activity, are what define MPM itself [29]. Therefore, the activation of STAT3 pathways may be more heavily influenced by epigenetic control. Combination therapies employing immune checkpoint inhibitors and STAT-3 inhibitors present a viable approach to long-term tumor management. Although further research is required, preclinical investigations and early clinical trials demonstrate its medicinal promise (Table 1). An interesting line of inquiry is the function of STAT-3 in modifying the tumor microenvironment, specifically by recruiting immunosuppressive cells [28,30].

### 1.4. Osteopontin (OPN)

Through its interactions with integrins and CD44 receptors, OPN, a matricellular protein, plays a role in tumor invasion, angiogenesis, and chronic inflammation in PM. These connections encourage the survival and spread of cancer cells by activating pathways like PI3K/Akt and FAK-Src-Rho. The chronic inflammatory response is further exacerbated by OPN, which is widely distributed in inflammatory tissues and promotes immune cell accumulation, macrophage retention, and cell survival activation. It is generally recognized that OPN is expressed in PM; a large number of studies have looked at this protein as a possible biomarker for diagnosis or prognosis; nevertheless, its exact function in malignant mesothelioma remains unclear [31,32]. The majority of research indicates that OPN contributes significantly to the development of cancer by promoting angiogenesis and the growth, invasion, and motility of tumor cells. OPN has been thoroughly investigated as a diagnostic biomarker for malignant mesothelioma, often in conjunction with mesothelin. An ELISA analysis of plasma or serum samples from PM patients revealed that OPN levels were higher in patients than in healthy donors or even in healthy people who had been exposed to asbestos. However, OPN’s low sensitivity and specificity have also raised doubts about its true clinical utility as an early diagnostic marker; for example, circulating levels of OPN were unable to distinguish between malignant and chronic inflammatory lung illnesses [21,31,33]. According to recent reports, OPN can attach itself to the ligand of the Inducible T-cell costimulatory (ICOS-L), which is another molecule. By attaching itself to ICOS, a costimulatory receptor produced on activated T-cells, ICOS-L, a member of the B7 family, maintains T-cell immunity and the antitumor response. Instead, in a mouse model of breast cancer, the binding of ICOS-L to OPN encouraged tumor metastasis. Malignant mesothelioma in humans also expresses ICOS-L. Therefore, disrupting this binding could be investigated as a novel therapeutic strategy for this new molecular partner of OPN as well. According to recent research, OPN-mediated signaling promotes an immunosuppressive milieu, which increases treatment resistance. OPN-targeted treatments are still in the experimental stage, despite preclinical research showing promise as a therapeutic target [34].

### 1.5. Mesothelin (MSLN)

Nearly 30 years ago, the glycosylphosphatidylinositol-anchored protein MSLN was identified in an attempt to identify novel surface targets for immunotherapy. On the cell surface of mesothelial cells of the pleura, pericardium, peritoneum, and tunica vaginalis, it is typically only found in trace levels. The physiological and biological functions of homeostatic MSLN are not well understood. Several solid tumors, particularly MPM of the epithelioid histological subtype, overexpress MSLN. Another mechanism may involve the PI3K/Akt pathway to protect cancer cells from drug-induced apoptosis [18,19]. Mesothelin protects cancer cells from TNF-α-induced apoptosis by rapidly stimulating Akt phosphorylation under PI3K activation, inhibiting the expression of pro-apoptotic factors, such as Bad and Bax, and promoting the expression of anti-apoptotic genes, such as Bcl-2 and Mcl-1 [35]. A study of the tissues of 38 PM patients revealed a correlation between worse survival rates and increased MSLN expression. High MSLN expression, however, was linked to prolonged survival rates in a larger study with 91 patients, and an even larger investigation with over 1500 MPM patient tissues came to the same conclusion [36,37]. MSLN’s significance in carcinogenesis has been investigated because of its substantial overexpression in PM. Mucin 16 (MUC16/CA125), which is expressed by PM cells and linked to the aggressiveness and progression of cancer, is known to bind to MSLN. Because MSLN can bind to MUC16 on other tumor cells, it has been demonstrated that the MSLN–MUC16 relationship is crucial for tumor cell adhesion and metastasis. When MSLN is knocked down in PM, stem cell and epithelial–mesenchymal transition (EMT) genes are downregulated, which reduces tumor development and metastasis in vivo. MSLN’s function in facilitating cancer invasion has been demonstrated by its association with matrix metallopeptidase 9 (MMP-9) expression at the invasive margins of malignancies [38,39,40]. Additionally, because MSLN downregulation can restore cell sensitivity to cisplatin treatment, MSLN has been linked to chemoresistance. Since its identification as a potentially effective therapeutic target, MSLN has been the subject of immunotherapy research. Area I (N-terminal area; residues 296–390), region II (residues 391–486), and region III (C-terminal region; residues 487–598) make up MSLN’s extracellular domain. The membrane-distal region (MDR), which binds to MUC16, is represented by region I. The MSLN MDR has emerged as the primary target for current immunotherapy approaches because of the role the MSLN–MUC16 relationship plays in tumor development [41]. To prevent steric impediment, new tactics are now focusing on other areas. An MSLN-targeted treatment that targeted region III exhibited greater activation and cytotoxicity than one that targeted region I, according to an in vitro investigation. This suggests that the effectiveness of MSLN-directed therapy may be significantly influenced by the MSLN target location. Chimeric monoclonal antibodies (amatuximab), antibody–drug conjugates (anetumab ravtansine, BMS-986148, and BAY2287411), immunotoxins (SS1P and LMB-100), a cancer vaccine (Listeria monocytogenes vaccine expressing MSLN), and CAR T cell immunotherapy are among the immunotherapy approaches that target MSLN in MPM. In conclusion, mesothelin uses the PI3K/Akt and MAPK pathways to promote tumor growth and immune evasion. More research is needed on mesothelin-targeted treatments in early-phase clinical trials (Table 1). PD-1 inhibitors and mesothelin-targeted strategies have demonstrated synergistic effects, considerably increasing overall survival in some patient subgroups [42].

### 1.6. Programmed Death-Ligand (PD-1/PD-L1)

Human PD-1 is a membrane protein belonging to the CD28 family, normally expressed by immune cells, including T and B lymphocytes, macrophages, and dendritic cells. Through its interaction with its ligand, PD-L1, it plays a key role in the negative regulation of immune responses. PD-1 can also be expressed on tumor-infiltrating lymphocytes (TILs), while tumor cells may express PD-L1 at varying levels, contributing to the inhibition of CD4+ and CD8+ T-cell activation and the apoptosis of antigen-specific T-cell clones [43,44].

PD-L1 expression, assessed through immunohistochemistry, appears to have a negative prognostic impact in various solid tumors. Approximately 20–50% of malignant pleural mesotheliomas (MPM) exhibit PD-L1 expression, considering a threshold of ≥1% positive cells. Higher PD-L1 expression levels are seemingly associated with worse clinical outcomes and are more frequently observed in sarcomatoid subtypes [45,46,47,48].

Over the past decade, monoclonal antibodies targeting programmed cell death protein 1 (PD-1) or its ligand PD-L1 have been globally approved for clinical use, either as monotherapy or in combination with chemotherapy, for the treatment of various malignancies, including thoracic cancers such as mesothelioma (Table 1). In mesothelioma, the most robust evidence supports their use in first-line and salvage therapy, while clinical trials investigating their role in neoadjuvant, adjuvant, and multimodal treatment approaches are still underway.

Ipilimumab and nivolumab together have recently been approved by the FDA and EMA as the new standard of therapy for unselected patients with unresectable MM in the first-line scenario [49]. It is currently unknown which mesothelioma patients benefit from immunotherapy and which do not, despite these encouraging findings. After 36 months, 28% of patients who responded to the ipilimumab–nivolumab combination in the Checkmate-743 trial were still responding. However, compared to 5% of patients treated with chemotherapy, 18% of patients treated with immunotherapy experienced primary refractories.

Other investigations have also documented the occurrence of early progression or even hyper-progressive illness in MM patients receiving ICIs. Furthermore, non-epithelioid patients benefit most from the combination of ipilimumab and nivolumab in a first-line scenario, perhaps because chemotherapy is inefficient for this histotype, while epithelioid patients did not experience the same degree of benefit. The CONFIRM study indicated that PD-1 inhibitors as monotherapy were better than a placebo in terms of OS and PFS in the second-third line of therapy, while the PROMISE-meso trial found that they were not better than chemotherapy (vinorelbine or gemcitabine) [50,51].

The PROMISE-meso and CONFIRM studies both documented improvements in epithelioid patients with ICIs. Specifically, in the PROMISE-meso study, the ORR was 22% for pembrolizumab-treated epithelioid individuals and 6% for chemotherapy-treated patients. Finding predictive biomarkers is therefore essential in light of these inconsistent outcomes and inconsistencies with the use of ICIs for the treatment plan of mesothelioma, particularly for patients with epithelioid disease, where the effectiveness of immunotherapy is less certain.

A number of initiatives are in progress to find predictive biomarkers of ICI response. In contrast to other malignancies, MPM patients’ response to ICIs of PD-L1 and TMB still has a poor and unclear prognostic utility. This is most likely because mesothelioma patients have a wide range of tumoral genetic heterogeneity and histological variations [50,52].

## 2. Genetic Mutations in MPM

### 2.1. BAP1

The BRCA1-associated protein-1 (BAP1) functions as a homologous recombination deoxyribonucleic acid (DNA) repair component of the BRCA1/BARD1 complex and is in charge of de-ubiquitinating histones, which in turn regulates protein transcription and the cell cycle. Mesothelioma is one of the cancers linked to pathogenic germline variants of *BAP1*; indeed, germline mutations in *BAP1* are responsible for 1–7% of malignant mesotheliomas [53,54]. Furthermore, somatic inactivating aberrations in *BAP1*, including point mutations, copy number loss, and rearrangements, are present in 20–64% of PM. The majority of genomic investigations conducted in PM have linked prolonged OS to the presence of either germline or somatic *BAP1* aberration [55,56,57]. The usefulness of *BAP1* mutations as possible predictive biomarkers and targets for different systemic therapies in PM is not well understood. It has been suggested that, like *BRCA2*-mutant ovarian cancer, *BAP1*-altered PMs are characterized by an exceptionally high sensitivity to platinum-based treatment [58]. This theory was based on the observation that PM cells carrying *BAP1* mutations are unable to effectively repair platinum-induced DNA cross-links [59,60]. Additionally, *BAP1*-modified PM may be vulnerable to poly (ADP-ribose) polymerase inhibitors (PARPi). Because of their inflammatory tumor microenvironment and increased immune signaling, mesotheliomas with BAP1 abnormalities may be able to predict long-term responses to immune checkpoint inhibitors (ICPi) [61]. Emerging evidence suggests that EZH2 inhibitors, such as Tazemetostat, may counteract BAP1-deficient tumors. Studies have also identified the interplay between BAP1 loss and enhanced susceptibility to DNA damage, suggesting a potential for synthetic lethality-based strategies (Table 2) [62,63].

### 2.2. CDKN2A

The most common cause of p16 protein inactivation and the most commonly observed chromosomal alteration in PM is the loss of CDKN2A. A small percentage of PM patients have been linked to hypermethylation of *CDKN2A* as the source of p16 expression loss. Although some studies only reveal deletion in one-fifth of instances, the frequency of *CDKN2A* deletion in PM has most frequently been found to range from 61 to 88% in primary tumors. The *CDKN2A* deletions found by fluorescence in situ hybridization (FISH) have been used for prognostication purposes and to differentiate between benign mesothelial proliferations on effusions or biopsy material and PM [64,65,66]. The most commonly documented chromosomal change in PM (61–88%) is the deletion of material in 9p21, mostly *CDKN2A*, which encodes for the tumor suppressors p16INK4A and p14ARF. Consequently, a *CDKN2A* copy number aberration, either homozygous (HD) or hemizygous deletion or monosomy, was present in the majority (84.1%) of our PM cases. Cell cycle progression mediated by CDK4/6 is inhibited by the tumor suppressor p16INK4A [66,67]. Clinical testing is being performed on CDK4/6 inhibitors, such as Abemaciclib (Table 2). According to recent studies, immune checkpoint blockade and CDK4/6 inhibitors may enhance the therapeutic effects [68,69].

### 2.3. NF2

*NF2* is found on chromosome 22q12 and was first identified as the cause of neurofibromatosis type II, a family cancer syndrome. The protein that NF2 encodes is called moesin-ezrin-radixin-like protein (merlin), which belongs to the Band 4.1 family of cytoskeletal linker proteins and is also referred to as neurofibromin 2 or schwannomin. The 70-kDa protein merlin has three different domains: an alpha-helical domain, a C-terminal domain, and the FERM (4.1, ezrin, radixin, and moesin) domain at the N-terminus. A total of 30% to 40% of pleural mesotheliomas have somatic NF2 mutations [70]. Apart from nonsense/missense mutations or small/large deletions with loss of heterozygosity, which lead to bi-allelic loss of function, mesotheliomas can also exhibit additional structural abnormalities, such as gene rearrangement that disrupts the NF2 region [70,71]. It is noteworthy that *NF2* mutations are more common in sarcomatoid mesothelioma than in epithelioid mesothelioma. Clonality of mesothelioma cells was discovered through recent genomic sequencing using multi-sampling of the same patient’s mesothelioma tissues. This demonstrated that *NF2*/22q loss is a late event, but *BAP1*/3p21 loss is an early event. Another study similarly found intra-tumor heterogeneity of *NF2* mutation in pleural mesothelioma, suggesting that *NF2* mutation is a late event that could lead to more aggressive phenotypes [71]. Genes of the Hippo pathway, which appears to be a significant signaling pathway regulated by merlin in mesothelial cells, are frequently inactivated in mesotheliomas in addition to NF2. Merlin controls the important signaling pathway known as the Hippo pathway. Numerous physiological processes, such as cell division, proliferation, organ growth, embryogenesis, tissue regeneration, and wound healing, are facilitated by the Hippo pathway [72]. Heart illness, lung disease, liver disease, renal disease, and cancer are among the conditions linked to the dysregulation of this system [72]. MST1 and MST2 kinases, SAV1 (also known as WW45), MOB1A/B, and LATS1 and LATS2 kinases are among the many molecules that make up the Hippo pathway. It is regulated by a number of signals and extracellular conditions, such as cell density, cell polarity, cell attachment, mechanical cues, and soluble factors [72]. MST1 and MST2 kinases phosphorylate (activate) LATS1 and LATS2 when the Hippo pathway is activated. LATS1/2 can also be phosphorylated by MAP4Ks. The transcriptional coactivators TAZ and YAP are major targets of LATS1/2 kinases. Phosphorylation of YAP/TAZ results in their degradation or retention in the cytoplasm. Activated YAP/TAZ increases the transcription of multiple key genes in mesothelioma cells, promoting the growth and spread of the malignancy [71,72]. Between 7% and 11% of mesothelioma cases have been found to have *LATS2* genetic abnormalities. Several Hippo pathway gene areas, including *MST1* and *LATS1*, showed a common allelic loss, according to a thorough genome analysis of mesothelioma tissues. These component genes’ promoter sequences have also been found to exhibit epigenetic changes [73]. Perhaps a more significant result is that asbestos-induced DNA damage or a direct inflammatory response are not the causes of *NF2* mutations. The lack of reports of an elevated risk of mesothelioma among carriers of *NF2* germline mutations supports this theory [74]. Other pathways are known to be potentially implicated in merlin inactivation, in addition to the genetic or epigenetic inactivation of NF2 itself. There have been reports of splicing variations in *NF2* mRNA, and the same study found that mesothelioma has higher levels of expression of the carboxyl-terminal version (isoform 2), which may not have a tumor suppressive function. Additionally, the overexpression of certain *NF2*-targeting microRNAs, such as has-miR-885-3p, appeared to decrease NF2 [75]. In summary, the Hippo signaling pathway is activated by the loss of NF2 activity, which encourages tumor growth and medication resistance. A novel treatment approach is to target downstream effectors like YAP/TAZ. TEAD inhibitors have the potential to reverse *YAP/TAZ*-driven oncogenesis and are presently undergoing early-phase studies (Table 2) [76].

## 3. MicroRNAs in Pleural Mesothelioma

MicroRNAs (miRNAs) are small non-coding RNA molecules that regulate gene expression post-transcriptionally, playing critical roles in cellular processes such as proliferation, apoptosis, and differentiation. In pleural mesothelioma, miRNAs have emerged as key regulators of tumor biology, contributing to oncogenesis, progression, and therapy resistance. Several miRNAs, including miR-31, miR-126, and miR-34a, have been implicated in PM (Table 3) [33].

### 3.1. miR-126

mir-126 is commonly downregulated in PM, which derepresses VEGF signaling and promotes angiogenesis and tumor growth. In experimental models, miR-126 restoration has been demonstrated to suppress tumor growth, indicating that it may be a promising therapeutic target. Most people agree that miR-126 is a tumor suppressor microRNA. It plays a role in blocking important stages in the development of cancer, including cell migration, invasion, and proliferation [77,78]. MiR-126 expression is frequently downregulated in malignant pleural mesothelioma, which promotes tumor development and spread. Poorer clinical outcomes and more aggressive illness have been linked to MPM cells’ downregulation of miR-126. miR-126 targets genes that are implicated in key signaling pathways linked to the development of cancer, including the EGFR (epidermal growth factor receptor) and VEGFR (vascular endothelial growth factor receptor) pathways. VEGFR has a role in angiogenesis, or the creation of new blood vessels, a critical mechanism for tumor growth and metastasis, and miR-126 inhibits VEGFR2. miR-126 helps to restrict the blood flow to tumors by targeting VEGFR2, which may stop tumor growth [78,79,80,81]. Additionally, EGFR, which is essential for fostering cell migration, survival, and proliferation in a variety of malignancies, including PM, can be targeted by miR-126. miR-126 prevents cancer cells from multiplying and invading nearby tissues by downregulating EGFR. miR-126′s capacity to inhibit tumor cell motility and invasion is one of its primary characteristics. Mesothelioma cells become more invasive and have the ability to spread to other tissues when miR-126 expression is lost. Cell migration involves integrins, which are proteins that aid in cell adhesion to the external matrix and to one another. miR-126 can control the expression of these proteins [79,80]. miR-126 helps stop mesothelioma cells from spreading into neighboring organs and developing into new tumors by suppressing the expression of integrin. Lower survival rates and a worse prognosis, including quicker disease progression, have been associated with reduced expression of miR-126 in PM. A more aggressive phenotype of mesothelioma that is more likely to spread and withstand treatment may be reflected in the downregulation of miR-126. As a result, miR-126 expression levels may be employed as a predictive biomarker in PM, aiding in the evaluation of the disease’s severity and the forecasting of patient outcomes. Restoring mesothelioma cells’ expression of miR-126 could provide a potential treatment approach [79,80]. It might be feasible to prevent angiogenesis, encourage tumor cell death, and suppress tumor cell invasion by raising miR-126 levels. miRNA mimics, which are synthetic compounds that imitate the function of miR-126, or gene therapy techniques could be used to deliver miR-126 to tumor cells. This might aid in reversing the hostile conduct linked to reduced miR-126 levels in mesothelioma. MiR-126 restoration in combination with other treatments, such as immunotherapy or chemotherapy, may increase the efficacy of those treatments and lead to better patient outcomes [82,83,84]. Understanding how miR-126 expression can be controlled and whether its restoration can result in successful PM treatments are the main goals of current research. Although preclinical research on the effects of miR-126 restoration in mesothelioma cells is still in its early phases, it has the potential to yield new therapeutic strategies that could increase survival rates and lessen the disease’s aggressiveness [82,83,84].

### 3.2. miR-31

By controlling cell cycle genes including *CDK4/6* and *p27*, mir-31 suppresses tumors; its absence is linked to more invasive tumors. A microRNA, or tiny non-coding RNA molecule, called miR-31 has been found to be an oncogene in a number of malignancies, including PM. It has a role in controlling the expression of several genes that can affect the invasion, migration, and proliferation of cancer cells. According to studies, miR-31 is frequently overexpressed in PM tissues, and this overexpression is linked to poorer prognosis and more aggressive tumor behavior. *P53* and *FAS* are two important tumor suppressor genes that miR-31 can target. It has the ability to downregulate these tumor suppressors, which increases cell survival and apoptosis resistance [85]. Furthermore, it has been demonstrated that miR-31 affects a number of signaling pathways, including the Wnt/β-catenin pathway, which is essential for controlling cell division and growth. Poor survival outcomes are correlated with high levels of miR-31 in PM tissues, indicating that miR-31 may be a useful biomarker for prognosis in patients with mesothelioma. A more aggressive tumor phenotype may be indicated by elevated miR-31 expression, and its existence may aid in the prognosis of the disease. miR-31 is being studied as a possible therapeutic target because of its part in tumor growth [86]. Researchers hope to reverse the carcinogenic consequences of miR-31 and restore the activity of tumor suppressor genes by blocking it. miR-31 inhibitors have demonstrated potential in preclinical research in slowing tumor development and making cancer cells more sensitive to chemotherapeutic treatments. To reduce the activity of miR-31 in PM cells, researchers are looking into ways to directly target it with small compounds or delivery mechanisms (such as nanoparticles). Given the poor prognosis and limited therapy choices currently available, miR-31 suppression in combination with other medications may improve the overall therapeutic response in PM [84,86].

### 3.3. miR-34a

The tumor suppressor gene *p53* regulates miR-34a, and this is one of its essential features. The transcription of miR-34a is activated by p53 in response to cellular stress or DNA damage. As a result, several target genes involved in cell cycle progression and survival are repressed, which encourages cell death and inhibits the growth of tumors. However, in PM, mutations or changes in the *p53* gene frequently cause disruptions in the p53 pathway, which can result in decreased expression of miR-34a. The poor prognosis and aggressiveness of mesothelioma are further exacerbated by this loss of miR-34a [87]. Numerous genes implicated in important pathways linked to cancer are targeted by miR-34a. One gene that prevents apoptosis is BCL-2. By directly targeting BCL-2, miR-34a encourages cancer cells to undergo apoptosis. SIRT1 is a gene that has a role in the survival and stress response of cells. When miR-34a inhibits SIRT1, tumor cell survival is decreased, and cell death is encouraged. The progression of the cell cycle is influenced by the genes CDK6 and E2F3. By suppressing their expression, miR-34a stops unchecked cell division and causes cell cycle arrest. MET encodes for a tyrosine kinase receptor implicated in the development and spread of tumors. By suppressing MET expression, miR-34a can stop the migration and invasion of cancer cells [87,88]. In PM, the downregulation of miR-34a is linked to worse clinical outcomes, such as a more aggressive disease phenotype and poor overall survival. It may be possible to use the levels of miR-34a in PM tissue samples as a prognostic biomarker to forecast how the illness will develop and if a patient will react to specific treatments [87]. In PM, miR-34a has drawn attention as a possible therapeutic target because of its tumor-suppressive characteristics. Restoring miR-34a expression in PM cells may aid in decreasing tumor development, sensitizing cells to chemotherapy, and reversing resistance to cell death [87]. The introduction of miR-34a mimics, which are synthetic molecules that imitate the effect of miR-34a, or the use of small molecules to induce miR-34a expression in tumor cells are two approaches that are being investigated for restoring miR-34a function [87]. miR-34a-based treatments are currently being tested in clinical trials for a number of malignancies, and the results may also be applicable to PM. To increase the efficacy of other treatments, miR-34a may be used in conjunction with them. Restoring miR-34a in PM cells, for instance, may improve the effectiveness of immunotherapy or chemotherapy by sensitizing the cells to these treatments. Treatment effects may also be enhanced by focusing on both miR-34a restoration and oncogenic microRNA inhibition (e.g., miR-31) [84].

In this manuscript, we have focused on miR-126, miR-31, and miR-34a due to their pivotal roles in regulating critical pathways involved in the pathogenesis of PM, such as tumor angiogenesis, proliferation, invasion, and apoptosis resistance. Their therapeutic potential, as discussed above, is supported by experimental data showing their involvement in various mechanisms of mesothelioma progression and their modulation by either restoration (miR-126, miR-34a) or inhibition (miR-31). However, several other factors and miRNAs warrant attention as potential therapeutic targets for mesothelioma:

miR-200 family: This family, including miR-200c, has been linked to the epithelial–mesenchymal transition (EMT), a process associated with metastasis and chemoresistance in mesothelioma. The downregulation of miR-200 leads to the activation of EMT, enhancing the migratory and invasive properties of mesothelioma cells. Therapeutic strategies aimed at restoring the expression of miR-200 could potentially reverse EMT and reduce metastasis, improving treatment outcomes in PM patients [89,90].

miR-155: miR-155 is often overexpressed in several cancers, including mesothelioma. It is involved in immune modulation and promotes inflammation, which could contribute to PM progression and immune evasion. Targeting miR-155 could potentially enhance the immune response against mesothelioma by reducing tumor-associated immune suppression [91].

miR-146a: Known for its role in regulating the immune response and inflammation, miR-146a has been shown to be downregulated in PM. Its restoration might improve the tumor microenvironment by controlling inflammatory cytokine levels and promoting immune surveillance [92] [93].

We have emphasized miR-126, miR-31, and miR-34a primarily because of their well-established roles in the regulation of fundamental cellular processes and their potential for clinical translation. Nonetheless, exploring these other factors and miRNAs could further expand our understanding of mesothelioma biology and provide additional therapeutic avenues. We believe that incorporating these additional targets into ongoing and future research efforts will be critical for improving the prognosis of PM patients.

## 4. Epigenetic Modifications

Tumor-suppressor genes may become inactive as a result of group-specific protein modifications (such as acetylation, methanation, and phosphorylation) and DNA modifications (such as methylation) that make up the epigenetic genome. Research has indicated that one of the primary causes of PM is epigenetic abnormalities (Table 4). The process of PM-mediated epigenetic modification is closely linked to a section of the cancer genome that is rich in dihydroxy acids and cytosine (C)-phosphate (P)-guanine (G) dinucleotides. These sections are known as CpG islands, and they are usually found in the promoter regions of genes and range in length from 300 to 3000 bp. DNA methylation can inhibit the activity of tumor-suppressor genes by methylating CpG islands. The development of PM is significantly influenced by DNA methylation [94,95,96]. The multi-domain structural protein known as ubiquitin-like with PHD and ring finger domains 1 (UHRF1) is principally in charge of attracting DNA methyltransferases to freshly produced DNA. It is abundantly expressed in a variety of malignancies, leading to group-specific protein changes and DNA methylation. According to Reardon et al., PM cells have significantly higher levels of UHRF1 mRNA and protein than normal leukocytes. Additionally, the expression level of UHRF1 was associated with a poorer OS and prognosis; patients with high UHRF1 expression had a median OS of only 8.7 months, whereas patients with low UHRF1 expression had a median OS of up to 22.2 months [97]. By creating a mouse model of PM, inhibitors of human double minute 2 (HDM2) or specificity protein 1 (SP1), like pucacilin, can reduce UHRF1 overexpression in PM mice by boosting p53 signaling, which in turn stops PM growth. Therefore, although clinical trials for UHRF1-targeted medicines have not yet been carried out, and further research is needed, targeting UHRF1 could greatly enhance patient survival [98]. By controlling cell proliferation, cell death, DNA repair, and self-renewal, K36 demethylase 4A (KDM4A) demethylates specific histone H3 lysine, which indirectly influences cell division and contributes to the development of cancer [99]. By preventing cell cycle functions and causing cell death, the KDM4A inhibitor slows the growth of tumor cells. KDM4A deficiency inhibited MPM cell proliferation and decreased cancer cell viability, according to Lapidot et al. Targeting KDM4A is still at the basic research stage at this time, and more research is required [100]. A class of methylase proteins known as lysine-specific demethylase 1 (LSD1) eliminates the monomethylation and dimethylation of histones H3, H4, and H9, which prevents target gene transcription and encourages the development of cancer. Since the epithelial–mesenchymal transition (EMT) is linked to the prognosis of PM and may be a factor in chemotherapy resistance, MPM treatment may benefit from focusing on EMT. According to Wirawan et al., LSD1 induces EMT through the FAK-Akt-GSK3β pathway; hence, blocking LSD1 could be a useful therapeutic approach [101].

## 5. Progress in Clinical Trials

While many therapeutic targets are still in preclinical stages, several clinical trials have yielded promising results (Table 5):

### 5.1. Tazemetostat (EZH2 Inhibitor)

Tazemetostat has demonstrated, although with limited overall efficacy, illness control in PM patients with BAP1 deficiency. The advancement of cancer is linked to the overexpression of the enhancer of zeste homolog 2 (EZH2). The carcinogenic polycomb repressive complex 2 component EZH2 is commonly found in conjunction with *BAP1* deletion. Researchers started a phase II trial to evaluate the impact of tazemetostat, an EZH2 inhibitor, in relapsed PM patients with inactivated BAP1 since mesothelioma cells with inactivated BAP1 are susceptible to EZH2 pharmacologic inhibition. At week 12 (the trial’s main result), the illness control rate was 54%, and at week 24, it was 28%. Additionally, Tazemetostat’s toxicological profile was excellent. Combining EZH2 inhibitors with conventional chemotherapy may improve response durability, according to early-phase research [102].

### 5.2. Rucaparib (PARP Inhibitor)

Rucaparib has demonstrated the ability to increase BAP1-negative patients’ progression-free survival. Rucaparib has been shown to be an effective treatment for pleural mesothelioma patients with either *BAP1* or *BRCA1* deletion at immunohistochemistry, according to recent data from the MIST-1 trial. More recent studies seek to investigate combinations with radiation therapy to take advantage of weaknesses in DNA repair [103,104].

### 5.3. Abemaciclib (CDK4/6 Inhibitor)

Abemaciclib decreased tumor burden in patients with p16INK4A deficiency, but there were few therapeutic advantages. Therefore, the goal of the current study was to assess the antitumor potential of combining CDK4/6 inhibitors, specifically the recently developed and promising medication abemaciclib, with the conventional chemotherapeutic drugs (cisplatin + pemetrexed) used to treat unresectable PM. Abemaciclib is distinct from other CDK inhibitors in that it targets CDK4/6 and also has some inhibitory effects on CDK9. Furthermore, due to its unique properties, it is the only inhibitor in this class that exhibits action against breast cancer and other solid tumors when used alone. The results show that in PM cell lines, the concurrent administration of abemaciclib, cisplatin, and pemetrexed had stronger antiproliferative effects than chemotherapy. Additionally, this concurrent therapy induces autophagic cell death in H28 cells and cytostatic effects in ZS-LP and MSTO-211H cells, resulting in senescence [105,106,107].

### 5.4. CheckMate 743

Nivolumab and ipilimumab were studied in the CheckMate 743 trial as a first-line therapy for PM that cannot be cured. A total of 605 individuals were randomly assigned to receive either conventional treatment with cisplatin or carboplatin and pemetrexed or nivolumab + ipilimumab in this open-label phase III research. The median OS after immunotherapy was 18.1 months, while the median for chemotherapy was 14.1 months (hazard ratio: 0.74; 95% CI: 0.60–0.91). The benefits for patients with non-epithelioid histology were especially noteworthy. Treatment results were unaffected by the expression of Programmed Death-Ligand 1 (PD-L1) [ClinicalTrials.gov Identifier: NCT02899299].

### 5.5. DREAM3R

In treatment-naïve PM patients, the DREAM3R phase III trial compares durvalumab with cisplatin/carboplatin and pemetrexed to chemotherapy alone. Programmed Death-Ligand 1 (PD-L1) is a bodily substance that is blocked by the antibody durvalumab, a type of human protein. By blocking PD-L1, the body’s immune system can more effectively combat cancerous cells. Durvalumab has been found in studies to reduce tumor size and halt tumor growth in certain cancer patients. Prior research has demonstrated that durvalumab and chemotherapy work well together for advanced mesothelioma. With OS as the major endpoint and progression-free survival (PFS), objective response rate (ORR), and quality of life as secondary objectives, this trial expands on phase II research. Recruitment aims to recruit 480 patients under a 2:1 randomization. We are waiting on the results [ClinicalTrials.gov Identifier: NCT04334759].

### 5.6. BEAT-Meso

The BEAT-Meso study compares bevacizumab with chemotherapy to atezolizumab and bevacizumab with conventional chemotherapy. Atezolizumab and bevacizumab, the two monoclonal antibodies utilized in this trial, were made in a lab. The purpose of atezolizumab is to bind to immune cells and increase their ability to fight cancer. The European Medicines Agency has authorized bevacizumab with atezolizumab for the treatment of lung cancer and other malignancies. OS is the main outcome, while PFS, ORR, and disease control at 24 weeks are secondary outcomes. There are 400 patients enrolled in 45 clinics around Europe [ClinicalTrials.gov Identifier: NCT03762018].

### 5.7. AtezoMeso

The AtezoMeso phase III trial compares adjuvant atezolizumab to placebo after perioperative chemotherapy and surgical resection. Disease-free survival is the main goal, and quality of life, OS, and safety are the secondary goals. For a maximum of 12 months, or until recurrence or intolerable toxicity occurs, therapy is given every 21 days [ClinicalTrials.gov Identifier: NCT04566637].

### 5.8. eVOLVE-Meso

Volrustomig, a PD-1/CTLA-4 bispecific monoclonal antibody, is evaluated in the eVOLVE-Meso study in comparison to chemotherapy or nivolumab and ipilimumab. This worldwide phase III trial started in November 2023 with OS as the main outcome. By 2028, results are expected [ClinicalTrials.gov Identifier: NCT06097728].

### 5.9. INFINITE

In PM that has already received treatment, the INFINITE phase III trial compares chemotherapy alone with adenovirus-delivered interferon-alpha-2b in combination with celecoxib and gemcitabine. Interferon alfa-2b, a virus that carries a gene alteration for the cancer cells, is administered as part of gene therapy. A viral vector is created when a virus introduces a gene into a different cell. Adenovirus cells designed to insert into the interferon protein gene are present in this treatment. Immune cells are drawn to the interferon release. T-cells and other immune system cells should be stimulated to target cancer cells as the therapy’s overall goal. Targeting tumor cells, the intrapleural treatment stimulates the immune system. OS is the main endpoint, and outcomes are anticipated by the end of 2024 [ClinicalTrials.gov Identifier: NCT03710876].

### 5.10. DENIM

In contrast to supportive care, the DENIM trial compares dendritic cell immunization with autologous cells loaded with tumor lysates as a maintenance treatment after chemotherapy. By exposing them to tumor antigens, dendritic cell (DC) therapy seeks to stimulate T-cell proliferation and encourage the activation of CD4+ and CD8+ T-cells, enabling CD8+ T-cells to penetrate the tumor microenvironment. By means of an ex vivo maturation process triggered by cytokines, DC can be obtained from the patient’s bone marrow or peripheral blood. Tumor antigens (peptides, lysate, and others) are loaded into mature DCs, processed by the cell, and then transported to the cell’s surface (by means of MHC I and II molecules). After processing, these DC cells are returned to the patient to boost the body’s defenses against the tumor. OS is the main endpoint. ORR, PFS, and survival rate at 18 months are examples of secondary endpoints [ClinicalTrials.gov Identifier: NCT03610360].

### 5.11. ATOMIC-Meso

In non-epithelioid PM, ATOMIC-Meso compares chemotherapy and pegargiminase, an arginine-depleting drug, to chemotherapy alone. Cancers that lack the urea cycle and tumor suppressor enzyme argininosuccinate synthetase 1 (ASS1) are inherently vulnerable to amino acid deprivation techniques and rely heavily on arginine for survival. With preclinical indications of single-agent action, pegylated arginine deiminase (ADI-PEG20; pegargiminase) breaks down arginine into citrulline and ammonia and causes cytotoxicity in a number of ASS1-silenced malignancies. Pegargiminase’s role in arginine metabolism was validated by the study’s longer OS (median: 9.3 months vs. 7.7 months) and PFS [ClinicalTrials.gov Identifier: NCT02709512].

### 5.12. LUME-Meso

In unresectable PM, the LUME-Meso study assessed nintedanib in conjunction with pemetrexed and cisplatin. Aimed at controlling tumor angiogenesis, growth, and metastasis of MPM, nintedanib is a twice-daily (bid) oral triple angiokinase inhibitor of vascular endothelial growth factor (VEGF) receptors 1–3, platelet-derived growth factor receptors α/β, and fibroblast growth factor receptors 1–3, as well as Src and Abl kinases. Progress-free survival (PFS) improved, but OS gains were not as strong, according to the results. Additional research is advised [ClinicalTrials.gov Identifier: NCT01907100].

### 5.13. PROMISE-Meso

In recurrent PM, the PROMISE-Meso study compared pembrolizumab to conventional treatment. Pembrolizumab did not significantly enhance OS; however, it did show a greater disease control rate [ClinicalTrials.gov Identifier: NCT02991482].

### 5.14. MesomiR 1

In PM patients, MesomiR 1 assessed TargomiRs (targeted microRNA-based treatment). Targeted minicells with a microRNA mimic are called targomiRs. They are made up of three parts: 1. A microRNA mimic based on miR-16: A variety of cancer types have been linked to the miR-16 family as a tumor suppressor. The mimic is a synthetic, double-stranded RNA molecule with 23 base pairs. 2. EDVs, drug delivery vehicles: EDVs are bacterial minicells, or nanoparticles, that are not alive. They serve as leak-proof micro-reservoir carriers that make it possible to package a variety of medications, proteins, and nucleic acids effectively. 3. The targeting component: An anti-EGFR bispecific antibody is used to target the EDVs to cancer cells that express EGFR. IVs are used to administer TargomiRs. According to preliminary findings, some patients had smaller tumors [ClinicalTrials.gov Identifier: NCT02369198].

### 5.15. HITOC

This trial assessed the impact of hyperthermic intrathoracic chemoperfusion (HITOC) after decortication and pleurectomy on overall survival and postoperative morbidity in patients with localized mesothelioma. While 25 consecutive patients underwent surgery followed by HITOC with cisplatin (125 mg/m^2^) administered for 70 min at 40–43 °C, 30 patients underwent surgery alone. An analysis was carried out on postoperative morbidity, complications associated with HITOC, and how HITOC affected survival. Compared to one case (3.3%) in the surgery group, the 30-day mortality rate in the HITOC group was 0%. Atrial fibrillation, renal impairment, and transient hypotension were among the temporary problems that occurred in 4 out of 25 (16%) patients in the HITOC group. The HITOC group’s progression-free survival was 8 months (95% CI 4.3–11.6), while the surgery-only group’s was 6 months (95% CI 2.5–9.9) (*p* = 0.79). The HITOC group’s total survival time was 28 months (95% CI 21.5–34.5), while the surgery-only group’s was 22 months (95% CI 17.5–26.5) (*p* = 0.75). An examination of risk factors for recurrence in the HITOC group revealed that early stages played a significant influence (*p* = 0.03). A safe treatment option that may increase survival for certain individuals with localized epithelial pleural mesothelioma is HITOC after pleurectomy and decortication [ClinicalTrials.gov Identifier: NCT05508555].

The combination of the PD-L1 inhibitor with chemotherapy or other treatment (antiangiogenic factors as an example) is being evaluated in different trials in treatment-naïve patients, and while the results are still pending, the strategy of combining immunotherapy with standard chemotherapy has already shown promise in other cancers. If successful, this could provide other effective strategies. Moreover, the combination of these therapies could have a synergistic effect, addressing both immune modulation and tumor growth, potentially leading to better outcomes in advanced mesothelioma. Other trials targeting the genetic landscape could open up new therapeutic avenues for patients with specific genetic mutations, offering a targeted treatment approach. However, only a limited number of patients could benefit from these strategies.

## 6. Conclusions

Pleural mesothelioma remains one of the most challenging malignancies to manage, with limited therapeutic options and a dismal prognosis. Despite incremental advances in treatment, current standard-of-care therapies have yielded only modest improvements in survival. Several critical gaps persist in the treatment of PM. Early detection remains a challenge since most patients are diagnosed at advanced stages due to the asymptomatic nature of early disease and the lack of effective screening tools. Therapeutic options are limited by the high degree of molecular heterogeneity and the absence of dominant oncogenic drivers in PM. These factors hinder the development of universally effective targeted therapies. Moreover, resistance to standard therapies, including chemotherapy and immunotherapy, poses a significant barrier, as mechanisms of therapeutic escape are poorly understood. Lastly, the disease’s aggressive biology, coupled with a lack of reliable prognostic and predictive biomarkers, limits clinicians’ ability to tailor treatments to individual patients, thereby reducing the efficacy of available interventions. A multifaceted strategy is crucial to extending the prognosis of PM patients. It will be essential to implement a customized medicine framework that uses molecular profiling to stratify patients and direct the choice of therapy. Moreover, techniques that combine immunotherapy, epigenetic modulators, and targeted medicines are expected to overcome resistance and produce synergistic results. The path forward in PM management requires addressing the complex interplay of genetic, epigenetic, and microenvironmental factors that drive the disease. By prioritizing early detection, leveraging precision medicine, and exploring innovative therapeutic strategies, the field has the potential to transform PM from a terminal diagnosis to a manageable condition. While significant challenges remain, ongoing research and emerging technologies provide a foundation for hope and progress in improving the lives of patients affected by this devastating disease.

## Figures and Tables

**Table 1:** Key Signaling Pathway Proteins and Their Role in PM.

Protein	Function	Mechanism of Action	Clinical Implications
Glucose-Regulated Protein 78 (GRP78)	Chaperone protein involved in protein folding and assembly	Regulates the Unfolded Protein Response (UPR) by interacting with PERK, ATF6, and IRE1; promotes tumor survival and chemoresistance by inhibiting caspases	Potential prognostic biomarker; target for GRP78 modulators such as BOLD-100, which disrupts calcium homeostasis and induces UPR-mediated cell death
Fibulin-3 (EFEMP1)	Extracellular matrix protein promoting tumor aggressiveness	Activates the PI3K/Akt pathway; knockdown reduces tumor burden and disrupts cell–ECM interactions	Investigated as a diagnostic and therapeutic target; function-blocking antibodies (mAb428.2) have shown promising tumor suppression effects
Signal Transducer and Activator of Transcription-3 (STAT-3)	Mediates immune evasion, inflammation, and proliferation	Activated by tyrosine phosphorylation (Tyr705); forms a complex with NFkB in chemoresistant PM cells	STAT-3 inhibitors combined with immune checkpoint inhibitors offer a potential therapeutic strategy
Osteopontin (OPN)	Matricellular protein involved in invasion, angiogenesis, and inflammation	Activates PI3K/Akt and FAK-Src-Rho pathways; contributes to chronic inflammation and immune cell retention	Potential diagnostic biomarker, but specificity is low; OPN-mediated signaling may promote immunosuppressive environments
Mesothelin (MSLN)	Surface glycoprotein overexpressed in PM	Binds to MUC16, facilitating tumor adhesion and metastasis; interacts with PI3K/Akt and MAPK pathways to promote survival and drug resistance	Target for immunotherapies including monoclonal antibodies (amatuximab), antibody-drug conjugates (anetumab ravtansine), and CAR T cell therapy
Programmed Death-Ligand (PD-1/PD-L1)	Immune checkpoint regulator	Inhibits T-cell activation and promotes immune evasion in PM	PD-1/PD-L1 inhibitors (nivolumab, pembrolizumab) approved for PM treatment; predictive biomarkers of response remain under investigation

Abbreviations: PM, pleural mesothelioma; UPR, unfolded protein response; ECM, extracellular matrix; NFkB, nuclear factor kappa B; PI3K, phosphatidylinositol 3-kinase; Akt, protein kinase B; MAPK, mitogen-activated protein kinase; FAK, focal adhesion kinase; CAR, chimeric antigen receptor; PD-1, programmed death-1; PD-L1, programmed death-ligand 1.

**Table 2:** Target genetic mutation in PM.

Gene	Primary Function	Alteration Frequency	Mechanism of Action	Therapeutic Implications
BAP1	DNA repair, transcription regulation, and cell cycle control	1–7% (germline), 20–64% (somatic)	Point mutations, copy number loss, rearrangements	Increased sensitivity to platinum-based therapy, potential target for PARP inhibitors (PARPi) and EZH2 inhibitors, possible response to immune checkpoint inhibitors (ICPi)
CDKN2A	Cell cycle regulation (encodes p16INK4A and p14ARF)	61–88%	Homozygous/hemizygous deletion (most common), promoter hypermethylation	CDK4/6 inhibitors (e.g., Abemaciclib), potential synergy with immune checkpoint blockade
NF2	Hippo signaling pathway regulation (encodes Merlin)	30–40%	Nonsense/missense mutations, deletions, rearrangements	Targeting YAP/TAZ within the Hippo pathway, TEAD inhibitors under clinical investigation

**Table 3:** MicroRNAs in Pleural Mesothelioma as Potential Therapeutic Approach.

MicroRNA	Mechanism of Action	Effects on PM	Potential Therapeutic Approach
miR-126	Suppresses VEGF and EGFR signaling, inhibiting angiogenesis and tumor proliferation.	Downregulated in PM, leading to increased tumor growth, migration, and invasion. Associated with poorer prognosis.	Restoration using miRNA mimics or gene therapy to inhibit angiogenesis and tumor spread. Potential combination with immunotherapy or chemotherapy.
miR-31	Regulates CDK4/6 and p27, controlling cell cycle progression. Can also downregulate p53 and FAS.	Overexpressed in PM, leading to increased tumor invasiveness, poor prognosis, and resistance to apoptosis.	Inhibition via miR-31 blockers to restore tumor suppressor gene function. Potential combination with chemotherapy to enhance therapeutic response.
miR-34a	Direct target of p53; regulates apoptosis (BCL-2), stress response (SIRT1), and cell cycle progression (CDK6, E2F3).	Downregulated in PM, leading to enhanced tumor cell survival, proliferation, and invasion. Correlated with poor survival.	Restoration using miRNA mimics or small molecules to induce miR-34a expression. Potential synergy with chemotherapy and immunotherapy.
miR-200 family	Inhibits epithelial-mesenchymal transition (EMT), reducing migratory and invasive tumor properties.	Downregulation leads to EMT activation, increasing metastasis and chemoresistance.	Restoration to reverse EMT and reduce tumor invasiveness.
miR-155	Modulates immune response and inflammation.	Overexpressed in PM, contributing to immune evasion and tumor progression.	Targeting miR-155 to enhance immune surveillance and reduce tumor-associated inflammation.
miR-146a	Regulates immune response and inflammatory cytokine levels.	Downregulated in PM, leading to an altered tumor microenvironment and reduced immune response.	Restoration to improve immune regulation and enhance anti-tumor immunity.

**Table 4:** Potential Therapeutic Approach in Epigenetic Modifications.

Target	Mechanism of Action	Effects on PM	Potential Therapeutic Approach
UHRF1 (Ubiquitin-like with PHD and Ring Finger domains 1)	Recruits DNA methyltransferases to newly synthesized DNA, leading to CpG island methylation and tumor-suppressor gene silencing.	Overexpressed in PM cells; associated with poor prognosis and reduced overall survival (OS).	Inhibition of HDM2 or SP1 (e.g., pucacilin) to restore p53 signaling and suppress UHRF1 overexpression.
KDM4A (K36 demethylase 4A)	Demethylates histone H3 lysine, regulating cell proliferation, DNA repair, and self-renewal.	Promotes PM progression by facilitating uncontrolled cell division.	KDM4A inhibition reduces tumor cell proliferation and viability.
LSD1 (Lysine-specific demethylase 1)	Removes monomethyl and dimethyl marks from histones H3, H4, and H9, repressing target gene transcription.	Induces epithelial-mesenchymal transition (EMT), contributing to chemotherapy resistance and poor prognosis.	LSD1 inhibition to block EMT via the FAK-Akt-GSK3β pathway.

**Table 5:** Progress in Clinical Trials.

Trial Name	Drugs Used	Phase	Clinical Trial Identifier	Trial Duration	Results
Tazemetostat Study	Tazemetostat (EZH2 inhibitor)	II	Not provided	12 weeks (primary endpoint)	Disease control rate: 54% (week 12), 28% (week 24). Well-tolerated toxicity profile.
MIST-1	Rucaparib (PARP inhibitor)	II	NCT03654833	Not provided	Study showed rucaparib was well tolerated, with grade 3–4 adverse events in 35% of patients. No treatment-related deaths. Common adverse events: nausea (69%), fatigue (54%), appetite loss (38%). Grade 3–4: respiratory infections (12%), anemia (12%). Dose reductions in 35% of patients.
Abemaciclib Study	Abemaciclib (CDK4/6 inhibitor), Cisplatin, Pemetrexed	Ib	NCT02079636	Not provided	Study assessed safety and efficacy of abemaciclib in combination therapy for advanced solid tumors, including NSCLC and mesothelioma. Well-tolerated with manageable adverse events.
CheckMate 743	Nivolumab, Ipilimumab	III	NCT02899299	Not provided	Median OS: 18.1 months (immunotherapy) vs. 14.1 months (chemotherapy). Non-epithelioid histology benefited most. PD-L1 expression had no impact.
DREAM3R	Durvalumab, Cisplatin/Carboplatin, Pemetrexed	III	NCT04334759	Ongoing	OS is the primary endpoint. Secondary: PFS, ORR, QoL. Recruitment target: 480 patients. Awaiting results.
BEAT-Meso	Bevacizumab, Atezolizumab, Chemotherapy	III	NCT03762018	Ongoing	OS is primary endpoint. Secondary: PFS, ORR, disease control at 24 weeks. 400 patients across 45 European clinics.
AtezoMeso	Atezolizumab vs. Placebo (adjuvant)	III	NCT04566637	Maximum 12 months	Primary endpoint: Disease-free survival. Secondary: OS, QoL, safety. Therapy every 21 days until recurrence or toxicity.
eVOLVE-Meso	Volrustomig (PD-1/CTLA-4 bispecific mAb), Chemotherapy, Nivolumab, Ipilimumab	III	NCT06097728	Expected results by 2028	OS is the primary outcome. Ongoing study.
INFINITE	Adenovirus-delivered interferon-alpha-2b, Celecoxib, Gemcitabine	III	NCT03710876	Expected results by 2024	OS is the primary endpoint. Intrapleural therapy stimulates immune system response. Awaiting final results.
DENIM	Dendritic cell immunization (tumor lysate-loaded autologous cells)	III	NCT03610360	Not provided	OS is primary endpoint. Secondary: ORR, PFS, 18-month survival rate. Dendritic cell therapy enhances immune response.
ATOMIC-Meso	Pegargiminase (ADI-PEG20), Chemotherapy	III	NCT02709512	Not provided	OS: 9.3 months (pegargiminase + chemo) vs. 7.7 months (chemo alone). PFS improved.
LUME-Meso	Nintedanib, Pemetrexed, Cisplatin	III	NCT01907100	Not provided	Improved PFS but OS benefit limited. Further studies recommended.
PROMISE-Meso	Pembrolizumab vs. Chemotherapy	III	NCT02991482	Not provided	No significant OS improvement, but higher disease control rate.
MesomiR 1	TargomiRs (microRNA-based therapy)	I	NCT02369198	Not provided	Some patients showed tumor size reduction.
HITOC	Hyperthermic Intrathoracic Chemoperfusion (HITOC) with Cisplatin	Not provided	NCT05508555	Not provided	OS: 28 months (HITOC) vs. 22 months (surgery alone). PFS: 8 months (HITOC) vs. 6 months (surgery alone). HITOC safe with manageable complications.
